# Editorial: Forward and inverse solvers in multi-modal electric and magnetic brain imaging: theory, implementation, and application

**DOI:** 10.3389/fnhum.2025.1629489

**Published:** 2025-06-10

**Authors:** Takfarinas Medani, Sampsa Pursiainen, Maria-Carla Piastra, Johannes Vorwerk, Richard M. Leahy

**Affiliations:** ^1^Ming Hsieh Department of Electrical and Computer Engineering, University of Southern California, Los Angeles, CA, United States; ^2^Mathematics, Computing Sciences, Tampere University, Tampere, Finland; ^3^Clinical Neurophysiology, Technical Medical Centre, Faculty of Science and Technology, University of Twente, Enschede, Netherlands; ^4^Biomedical Engineering Group, Department of Mechatronics, University of Innsbruck, Innsbruck, Austria; ^5^Institute of Measurement and Sensor Technology, UMIT TIROL - Private University for Health Sciences and Health Technology, Hall in Tirol, Austria

**Keywords:** EEG/MEG, sEEG/ECoG, forward and inverse analyses, modeling, simulation, sensitivity analysis

Understanding the complexities of brain function and structure remains one of the most challenging and exciting frontiers in neuroscience (Sporns, 2013). Advances in multi-modal brain imaging, integrating electrophysiological and magnetophysiological techniques such as non-invasive electroencephalography (EEG), invasive electroencephalography (iEEG), including both stereo-electroencephalography (sEEG), electrocorticography (ECoG), and magnetoencephalography (MEG), have provided unprecedented insights into neural dynamics (Hämäläinen et al., 1993; Gross et al., 2001; Baillet et al., 2001). A central challenge in this domain is solving the forward and inverse problems, which enable the estimation of the brain activity underlying measured signals (Baillet et al., 2001; Wolters et al., 2004; Mosher et al., 1999; Wolters et al., 2006; Piastra et al., 2021; Brette and Destexhe, 2012; Pursiainen et al., 2011).

This Research Topic aimed to bring together cutting-edge research on the theoretical foundations, computational methodologies, and practical applications of forward and inverse solvers in multi-modal electric and magnetic brain imaging. The contributions within this Research Topic span fundamental methodological advances, novel algorithmic frameworks, and state-of-the-art applications that push the boundaries of neuroimaging.

## Theoretical and computational advances

One of the significant challenges in EEG/MEG source analysis is accounting for the uncertainties in tissue conductivity. Vorwerk et al. investigated the global sensitivity of EEG source analysis to tissue conductivity uncertainties, emphasizing the importance of accurate head volume conductor models in forward problem solutions (Vorwerk et al., 2014, 2019; Lucka et al., 2012). Their findings highlight how variations in tissue conductivity can significantly impact source localization accuracy, concluding that an accurate parametrization of the head volume conductor model is as important as an accurate representation of the geometry (Wolters et al., 2006; Vorwerk et al., 2014, 2019). Piastra et al. conducted a validation study using stereotactic sEEG data to assess the accuracy of head volume conductor models. Their work underscores the necessity of precise modeling in achieving reliable source localization in combination with empirical validations. Erdbrügger et al. introduced the CutFEM forward modeling approach for EEG source analysis (Miinalainen et al., 2019; Vorwerk et al., 2017; Medani et al., 2015; Dubarry et al., 2023). This method offers enhanced flexibility in handling complex geometries and tissue interfaces in head volume conductor models, contributing to more accurate forward solutions in EEG studies.

## Algorithmic innovations and implementations

Advancements in algorithmic frameworks are crucial for improving source localization techniques. Luria et al. presented the SESAMEEG package, a probabilistic tool for source localization and uncertainty quantification in MEG/EEG analysis (Wipf and Nagarajan, 2009; Aydin et al., 2015; Mosher and Leahy, 1998). This package facilitates more robust and interpretable source estimates by incorporating probabilistic modeling. Ghosh et al. developed the Structured Noise Champagne algorithm, an empirical Bayesian approach for electromagnetic brain imaging that accounts for structured noise. This method enhances the reliability of source reconstructions in the presence of complex noise structures (Baillet et al., 2001; Mosher et al., 1999). Giri et al. proposed an F-ratio-based method for estimating the number of active sources in MEG data. Their approach aids in determining the optimal model complexity, balancing the trade-off between model fit and overfitting (Adler et al., 2022).

## Applications and future directions

The practical impact of these methodological advancements has been demonstrated in numerous studies. Guillen et al. explored optimized high-definition transcranial direct current stimulation (tDCS) in patients with skull defects and implants, demonstrating the clinical relevance of accurate modeling in neuromodulation therapies (Carla Piastra et al., 2021; Rampersad et al., 2014; Armonaite et al., 2025; Pursiainen et al., 2018). Huang visualized interferential stimulation of human brains, providing insights into the spatial distribution of electric fields during stimulation protocols (Huang et al., 2018; Saturnino et al., 2019). This work contributes to the optimization of stimulation parameters for therapeutic interventions. Furthermore, Prieto et al. analyzed the effects of lattice layout and optimizer selection on generating optimal transcranial electrical stimulation (tES) montages using the metaheuristic L1L1 method. Their findings inform the design of more effective stimulation configurations.

Collectively, these contributions underscore the importance of integrating theoretical, computational, and practical perspectives in advancing EEG/MEG source analysis as well as tES modeling. Future research directions may include the development of standardized pipelines (Vorwerk et al., 2014; Rampersad et al., 2014; Erdbrügger et al., 2024; Medani et al., 2023; He et al., 2020), the incorporation of machine learning techniques for model selection and parameter estimation, and the expansion of open-source tools to facilitate broader accessibility and reproducibility in the field (He et al., 2020; Vorwerk et al., 2018; Oostenveld et al., 2011; Tadel et al., 2011, 2019; Gramfort et al., 2013; Delorme and Makeig, 2004; Schrader et al., 2021; Cui et al., 2024).

We extend our sincere gratitude to all authors for their high-quality contributions and to the reviewers for their thoughtful feedback. We hope this Research Topic serves as a valuable resource for both newcomers and experienced researchers aiming to advance the state of the art in neuroelectromagnetic modeling.
